# Interchromosomal Duplications on the *Bactrocera oleae* Y Chromosome Imply a Distinct Evolutionary Origin of the Sex Chromosomes Compared to *Drosophila*

**DOI:** 10.1371/journal.pone.0017747

**Published:** 2011-03-07

**Authors:** Paolo Gabrieli, Ludvik M. Gomulski, Angelica Bonomi, Paolo Siciliano, Francesca Scolari, Gerald Franz, Andrew Jessup, Anna R. Malacrida, Giuliano Gasperi

**Affiliations:** 1 Department of Animal Biology, University of Pavia, Pavia, Italy; 2 Entomology Unit, FAO/IAEA Agriculture and Biotechnology Laboratory, Joint FAO/IAEA Programme, International Atomic Energy Agency, Vienna, Austria; National Institute on Aging, United States of America

## Abstract

**Background:**

Diptera have an extraordinary variety of sex determination mechanisms, and *Drosophila melanogaster* is the paradigm for this group. However, the *Drosophila* sex determination pathway is only partially conserved and the family Tephritidae affords an interesting example. The tephritid Y chromosome is postulated to be necessary to determine male development. Characterization of Y sequences, apart from elucidating the nature of the male determining factor, is also important to understand the evolutionary history of sex chromosomes within the Tephritidae. We studied the Y sequences from the olive fly, *Bactrocera oleae*. Its Y chromosome is minute and highly heterochromatic, and displays high heteromorphism with the X chromosome.

**Methodology/Principal Findings:**

A combined Representational Difference Analysis (RDA) and fluorescence *in-situ* hybridization (FISH) approach was used to investigate the Y chromosome to derive information on its sequence content. The Y chromosome is strewn with repetitive DNA sequences, the majority of which are also interdispersed in the pericentromeric regions of the autosomes. The Y chromosome appears to have accumulated small and large repetitive interchromosomal duplications. The large interchromosomal duplications harbour an *importin-4*-like gene fragment. Apart from these *importin-4*-like sequences, the other Y repetitive sequences are not shared with the X chromosome, suggesting molecular differentiation of these two chromosomes. Moreover, as the identified Y sequences were not detected on the Y chromosomes of closely related tephritids, we can infer divergence in the repetitive nature of their sequence contents.

**Conclusions/Significance:**

The identification of Y-linked sequences may tell us much about the repetitive nature, the origin and the evolution of Y chromosomes. We hypothesize how these repetitive sequences accumulated and were maintained on the Y chromosome during its evolutionary history. Our data reinforce the idea that the sex chromosomes of the Tephritidae may have distinct evolutionary origins with respect to those of the Drosophilidae and other Dipteran families.

## Introduction

Diptera are a group with an extraordinary variety of sex determination mechanisms [1]. Sex determination in *Drosophila* has been the paradigm for this group [2]. However, it is now known that only parts of the *Drosophila* sex determination pathways are conserved in other insects and the Tephritidae family affords an example. This family is well known for its prolific speciation and it includes some of the world's most significant agricultural pests [3]. The four major genera, *Anastrepha*, *Bactrocera*, *Ceratitis* and *Rhagoletis*, include pest species of remarkable economic importance. In particular, the genera *Bactrocera* and *Ceratitis* contain primarily Afrotropical, Australian, Mediterranean and Oriental highly invasive species. The karyotypes of all the species analyzed to date consist of six pairs of chromosomes including one pair of heteromorphic, highly heterochromatic sex chromosomes (XY), with the male being the heterogametic sex [4], [5], [6], [7]. The Y chromosome in the Tephritidae, despite being very variable in size, not only at the inter-specific but also at the intra-specific level, is postulated to be necessary to determine the development of male individuals [8]. The sex-determination molecular mechanism of the species belonging to this family differs from that of *Drosophila melanogaster*, whose Y chromosome is dispensable in the sex determination process but contains genes essential for male fertility [9]. In fact, based on the model formulated for the most studied tephritid species, *Ceratitis capitata* (medfly), a postulated Y-linked male-determining factor (M) directly or indirectly blocks the regulatory functions of key sex determination genes [2], [8]. Thus far, attempts to isolate Y-specific genes/sequences have resulted in the isolation of a Y-specific repetitive sequence in the medfly [10], [11], but the molecular nature of the M factor remains unknown.

Characterization of Y-specific sequences, apart from elucidating the molecular nature of the male determining factor, is also of crucial importance to characterize the molecular nature of the sex chromosomes and their evolutionary history within this dipteran family. Indeed, the nascent interest in understanding the evolutionary history of tephritid Y chromosomes is explained by the fundamental differences between the sex-determination molecular cascades between the Drosophilidae and Tephritidae. The fundamental question is whether it is possible to infer in tephritid flies a model of Y evolution applicable to a male-determining factor-carrying chromosome.

To date no sequenced genome is available for any tephritid species. Only from *Ceratitis capitata* have Y-specific and Y-enriched sequences been isolated [10], [11]. The Y chromosome of this species is largely degenerate with many repetitive sequences and it appears to be shaped by the massive accumulation of transposable elements [12]. No factors affecting fertility were detected outside the male determining region of the medfly Y chromosome [8].

We chose to investigate the Y chromosome sequence content of a tephritid species from the *Bactrocera* genus, *Bactrocera oleae* (olive fly). The olive fly is a very economically important species that has a major impact on global olive production [13]. Its Y chromosome is very small, dot-shaped and highly heterochromatic, and displays very high heteromorphism with the X chromosome [6], [14]. Moreover, in this species, the Y-based male determining system has been investigated at the molecular level [15], [16]. Given the difficulties involved in the analysis of heterochromatic sequences, we employed a Representational Difference Analyses (RDA) approach [17] to study the Y content of *B. oleae*. This technique has facilitated the isolation of female-specific sequences from *Schistosoma mansoni* [18], Y-specific sequences from *Silene latifolia* [19] and X- and Y-specific sequences from *Marchantia polymorpha* [20]. The data shows that the *B. oleae* Y chromosome appears to have accumulated small and large repetitive interchromosomal duplications that are not shared with the X chromosome, suggesting molecular differentiation between these two chromosomes. The isolated Y-specific and Y-enriched sequences were used to assess the presence of sequence conservation within several Tephritidae species and derive an hypothesis regarding the evolution of the sex chromosomes in this family.

## Materials and Methods

### Flies

Samples of *B. oleae* from the Demokritos and Israel Hybrid strains and from two natural populations, collected in two Eastern Meditteranean regions , Lebanon and Jordan, were considered in this study. The two laboratory strains were obtained from the FAO/IAEA Agriculture and Biotechnology Laboratory (Seibersdorf, Vienna, Austria). The Demokritos strain was originally set up with wild olive flies collected in Marathon, Greece, in 1966 and it has since been maintained on artificial rearing medium [21]. Wild male and female flies were added occasionally to maintain variability within the strain. The Israel Hybrid strain originated in 2006 by four back crosses of Demokritos females with wild males collected in Israel.

The wild Eastern Mediterranean samples were collected in 2005 from the locality of Jbeie (Republic of Lebanon) and in 2004 from the region of Salt (Hashemite Kingdom of Jordan).

### Genomic DNA preparations

Genomic DNA from wild and laboratory strain samples was individually extracted from male and female adult flies using the method previously used for *C. capitata* [22]. Following treatment with RNase A, the DNA was extracted with phenol/chloroform, precipitated with ethanol and resuspended in TE buffer (10 mM Tris-HCl, pH 8, 1 mM EDTA). The DNA concentration was quantified using a Nanodrop ND-1000 spectrophotometer (Nanodrop Technologies Inc., Wilmington, DE, USA).

### Representational difference analysis (RDA)

The Representational Difference Analysis (RDA) [17], [23] was applied to identify Y-specific or Y-enriched sequences in *B. oleae*. Briefly, to obtain maximum representation, male and female genomic DNA, extracted from twenty adults from the Israel Hybrid strain, was digested with four-cutter endonucleases, a CG-rich region cutter (*Msp*I) and an AT-rich region cutter (*Mse*I). Two pairs of each of the R, J and N series of adaptors (Table S1) were designed modifying adaptor-sequences from previously published protocols [17].

As a first step, the RDA protocol foresees the generation of male and female Representations by PCR of R-adaptors-ligated genomic fragments. To determine the optimum amount of input DNA, analytical amplifications were performed using serial dilutions of linker-ligated DNA: spanning four orders of magnitude (from 1000 to 0.064 pg/µl). Based on the results of this preliminary experiment, we chose concentrations of 8 pg/µl and 200 pg/µl to generate, respectively, the *Msp*I- and *Mse*I- male and female Representations. After the generation of Representations, adaptors were removed from the Representations by digestion, followed by spin-column purification (Purelink PCR Micro Kit, Invitrogen, Carlsbad, CA, USA). To generate the tester, J adaptors were ligated only to the Representations obtained from the male genomic DNA and control PCR reactions were performed to verify the adaptor substitution. In the subtractive hybridization, the Representations obtained from the female genomic DNA were used as driver DNA, using a 1/100 tester/driver ratio. After subtractive hybridization, the male-specific DNA was re-amplified to generate the Differential Product 1 (DP1) using J adaptors as primers. Following this procedure, a second and a third round of subtractive hybridizations were performed: the Differential Product 2 (DP2) was obtained using a new DNA tester generated by substituting the DP1 J adaptors for N adaptors and a 1/800 tester/driver ratio, while the Differential Product 3 (DP3) was obtained substituting the DP2 N adaptors for J adaptors and a 1/40000 tester/driver ratio. The final DP3 was cloned and sequenced to identify Y-specific and Y-enriched sequences in *B. oleae*.

### Cloning, sequencing and analysis of Differential Product 3 (DP3) sequences

The male specific bands present in the Differential Product 3 (DP3) were gel eluted and cloned into the PCR®2.1-TOPO® vector using the TOPO TA cloning kit (Invitrogen). Positive colonies were selected and the size of the insert quantified by *Eco*RI digestion and gel electrophoresis. Clones were sequenced using an ABI-310 automatic sequencer and the ABI Prism BigDye Terminator Cycle Sequencing Ready Reaction Kit v. 3.1 (Applied Biosystems, Foster City, CA, USA). Sequence analysis was performed using the BLAST family of programs from the National Centre for Biotechnology Information [24].

### PCR amplifications of DP3 sequences from male and female genomic DNA

To assess whether the sequences found in the two DP3s were male-specific, male and female genomic DNA from twenty *B. oleae* individuals of the Israel Hybrid strain were amplified using primers designed on DP3 sequences. The primers were designed using Primer3 [25] (Table S2) and PCR amplifications were performed in 10 µl reaction volumes using ∼10 ng *B. oleae* male and female genomic DNA, 1.5 mM MgCl_2_, Reaction Buffer (10 mM Tris, 50 mM KCl; pH 8.3), 0.2 mM dNTPs mixture, 10 pmol of each primer and 1 unit *Taq* DNA polymerase (Invitrogen). Amplification was achieved on an Eppendorf Mastercycler Gradient using the following program: an initial denaturation step at 94°C for 3 min; 30 cycles of denaturation at 94°C for 30 s, annealing at the primer-specific annealing temperature (Table S2) for 30 s, extension at 72°C for 45 s, followed by a final extension at 72°C for 5 min. Established primers and cycling conditions were used to amplify the *Botransformer* sequence [16]. The PCR products were analyzed on 14×10 cm 1.5% agarose gel slabs in 1×TAE buffer together with a 100 bp DNA ladder standard (Invitrogen). Ethidium bromide staining and exposure to UV light was used to visualize the bands.

Bands amplified using primers designed on DP3 sequences were directly cloned into the PCR®2.1-TOPO® vector (Invitrogen) and sequenced. Putative gene structures were predicted using the GenScan program [26]. Nucleotide sequences were aligned using T-coffee [27] and the neutral versus positive selection of synonymous and nonsynonymous substitutions of putative protein-coding fragments was assessed using the codon-based Fisher's exact test (Nei-Gojobori method) in MEGA4 [28].

### Southern hybridization analyses

To determine the *B. oleae* male and female genomic distribution of the DP3 sequences, genomic DNA from individual male and female flies (4 µg) from the Israel Hybrid strain were digested with *Msp*I or *Mse*I endonucleases. The digested DNAs were electrophoresed on a 20×14 cm 1% agarose gel in 1×TBE buffer and transferred to a positively charged nylon membrane according to Southern [29]. The membrane was hybridized at 55°C with 200 ng of probe DNA labelled with the Gene Images Alkphos Direct labelling system (GE Healthcare, Little Chalfont, UK) using the random primer method. The hybridization and detection protocols were those described by the manufacturer. Signal detection was performed using CDP-star followed by exposure to autoradiographic film (X-OMAT AR, Kodak).

### Chromosome preparation and fluorescence *in situ* hybridization

To assess the chromosomal distribution of the DP3 sequences, mitotic chromosome spreads were obtained using third instar larvae [6] of the *B. oleae* Israel Hybrid strain. Briefly, brain tissue was incubated in 1% sodium citrate for 10 min at room temperature and transferred to methanol-acetic acid 3∶1 solution for 4 min. The material was disrupted in 100 µl 60% acetic acid and dropped onto clean slides and dried. Fluorescence *in situ* hybridization (FISH) was performed using the following protocol; pre-hybridization was performed according to [8]: chromosome preparations were denatured at 80°C for 2 h, dehydrated by immersed in 30%, 50%, 70% and 96% ethanol for 2 min and allowing them to air dry. Slides were then immersed in NaOH 0.07 M for 2 min, rinsed for a few seconds in 0.4×SSC buffer plus 0.1% Tween 20 and dehydrated using ethanol as previously done. To generate the labelled probe, 1 µg of DNA resuspended in ddH_2_O (16 µl) was denatured by boiling for 10 min. 4 µl of labelling mix (Biotin High Prime kit; Roche, Basel, Switzerland) were added and the reaction was incubated over night at 37°C. After the reaction was stopped, ddH_2_O (5 µl), 20×SSC buffer (25 µl) and formamide (50 µl) were added and 25 µl of denatured probe was placed on each pre-treated slide. The hybridization was performed at 37°C over night in a humid chamber and detection of hybridization signals was performed using the Vectastain ABC elite kit (Vector Laboratories, Burlingame, CA, USA) and Alexa Fluor 594 Tyramide (Invitrogen). Chromosomes were DAPI stained and the slides were mounted using the VECTASHIELD mounting medium (Vector Laboratories). Chromosomes were screened under an epifluorescence Zeiss Axioplan microscope and images were captured using an Olympus DP70 digital camera.

## Results

### Identification and characterization of Y-specific or Y-enriched RDA sequences

**Figure 1:**
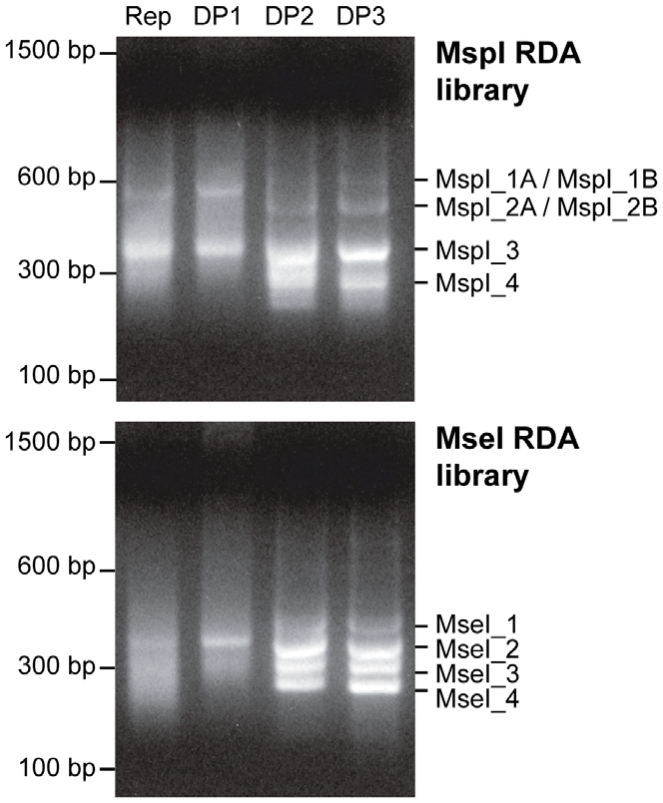
Agarose gel electrophoresis of *Msp*I (upper panel) and *Mse*I (lower panel) difference products from *B. oleae* Israel Hybrid strain genomic DNA. The Representation and the three Differential Products (DP1, DP2 and DP3) are shown.

The amplification patterns generated during each step of the RDA analyses are shown in Fig. 1. The final Difference Product 3 (DP3) of both *Msp*I and the *Mse*I RDA libraries each contained four discrete bands that were isolated by gel electrophoresis, gel-eluted, cloned and sequenced (Genbank acc. n. HR714291–HR714300).

From these eight bands, ten sequences were identified, as both the MspI-1 and the MspI-2 clones comprised two different sequences (MspI-1A and MspI-1B, MspI-2A and MspI-2B). All the sequences were analyzed using BLAST and the results are reported in Table 1.

**Table 1:** BLAST analyses of the RDA clones identified in this study.

Clone	Clone length (bp)	BLASTX^X^ or BLASTN^N^ best hits	alignment length	e-value	maximum identity (%)
MspI-3	315	No significant hit	*-*	*-*	*-*
MspI-4	246	No significant hit	*-*	*-*	*-*
MspI-1A	612	*Drosophila erecta* inactive *mariner* mobile element^N^	210	3E-30	75
MspI-2A	519	*Drosophila buzzatii* Osvaldo retrotransposon^N^	162	2E-13	71
MspI-2B	423	*Bactrocera oleae ovoA* (AJ535756.1)^N^	67	3E-04	80
		*Bactrocera oleae ovoA* (AJ535756.1)^N^	67	3E-04	80
MseI-1	398	*Bactrocera oleae ovoA* (AJ535756.1)^N^	57	7E-11	89
		*Bactrocera oleae ovoA* (AJ535756.1)^N^	57	7E-11	89
MseI-2	310	*Bactrocera oleae ovoA* (AJ535756.1)^N^	246	3E-71	84
		*Bactrocera oleae ovoA* (AJ535756.1)^N^	246	3E-71	84
MspI-1B	572	*Anopheles gambiae* AGAP006942-PA^X^	68	2E-14	55
MseI-3	283	*Anopheles gambiae* AGAP006942-PA^X^	52	1E-04	42
MseI-4	226	*Anopheles gambiae* AGAP006942-PA^X^	79	6E-11	48

The MspI-3 and the MspI-4 sequences presented no significant similarity (BLASTN and BLASTX) with any of the sequences present in Genbank. On the contrary, MspI-1A and the MspI-2A harbour sequences that are similar to known transposable elements (TEs). The fragment present in MspI-1A is similar to the transposase coding sequences of a *mariner* element of the *mellifera* family, [12], [30]; however, the presence of stop codons in the conceptual translation suggests that it may be an inactive element. MspI-2A contains a sequence similar to the *Osvaldo* retrotransposon first identified in *Drosophila buzzatii* [31], [32].

MspI-2B, MseI-1 and MseI-2 showed sequence identity with two different promotor regions present in the zinc-finger transcription factor encoding gene, *Ovo*, of *B. oleae* which codes for two alternative transcripts *OvoA* and *OvoB* with separate promotors [33] (Fig. S1A). Moreover, the MseI-1 and MseI-2 sequences are partially similar to the same section of the promoter region upstream of the *OvoA* transcript start site (nucleotide identity: 89% and 84%), as they share an identical 57 bp region. MspI-2B differs from the previous two sequences and shares partial identity with a different section of the promoter region upstream of the *OvoA* transcript start site (nucleotides identity: 80%).

Finally, the MspI-1B, the MseI-3 and the MseI-4 clones shared amino-acid similarity with Importin-4 proteins of many insect species. In particular, these three sequences shared similarity, in part or over their full length (such as MseI-4, 226 bp), to different portions of the *Anopheles gambiae* Importin-4 protein sequence (AGAP006942) (Fig. S1B). MspI-1B is similar (amino-acid identity: 55%) to the importin-beta domain, while MseI-4 to the N-portion of the Armadillo domain (amino-acid identity: 42%) and MseI-3 to the HEAT-repeated domain (amino-acid identity: 48%).

### Genomic distribution of the isolated RDA sequences

To investigate whether the transposable element-related sequences present in MspI-1A and the MspI-2A clones are Y-specific or Y-enriched sequences, PCR primers were designed (Table S2) and used to amplify male and female genomic DNA from the Israel Hybrid strain. For both the *mariner* and *Osvaldo* sequences, primer pairs produced the same amplification product in both male and female samples and both amplicons were of the expected size. Furthermore, the Southern blot analysis demonstrated that multiple copies of these sequences are spread throughout the *B. oleae* genome (not shown).

**Figure 2:**
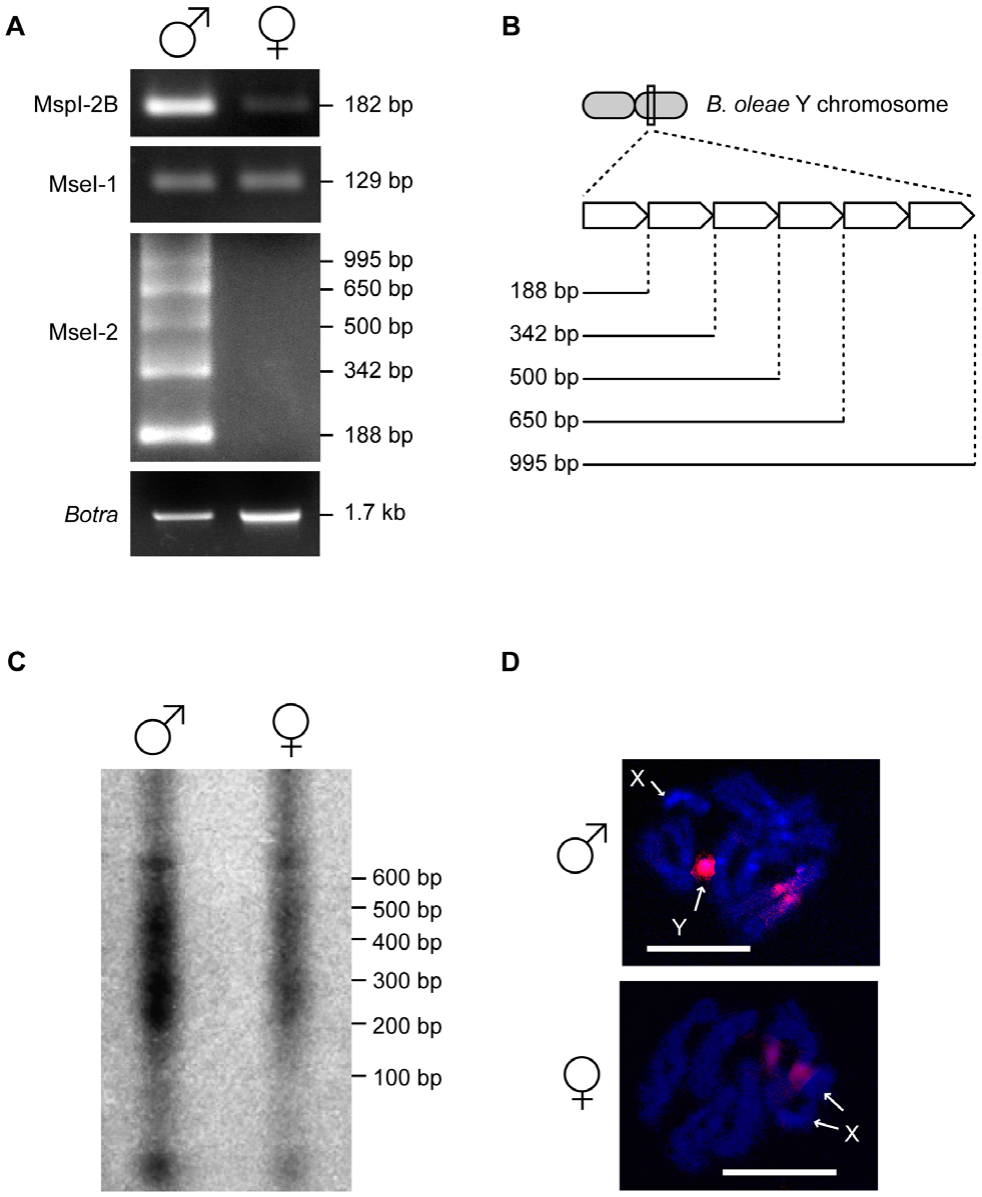
The *B. oleae* Y chromosome contains small duplicated regions similar to a portion of the *Ovo* promoter region. A) PCR amplifications on male and female genomic DNA from the Israel Hybrid strain using primers designed on the MspI-2B, MseI-1 and MseI-2 RDA clones. As a control, *Botransformer* (*Botra*) was amplified from the same samples. B) Schematic representations of the tandem repeat arrays, which are present on the *B. oleae* Y chromosome. The 188 bp repeat unit was isolated from the MseI-2 RDA clones. C–D) The enrichment of MseI-2-like sequences on the *B. oleae* Y chromosome was verified using Southern blot analyses (C) and fluorescence in situ hybridization (D) to male and female mitotic chromosomes preparations (scale bar 15 µm). For the Southern blot analyses male and female genomic DNA was digested with *Mse*I.

Primers were designed also on the *Ovo*-related sequences present in MspI-2B, MseI-1 and MseI-2 clones, and used to amplify male and female genomic DNA from the Israel Hybrid strain (Fig. 2A). Specifically, for MspI-2B the same amplification product was obtained in both male and female samples and both amplicons were of the expected size (182 bp). However, despite using the same amount of genomic DNA template, the intensity of the male product was much greater than that of the female. Also the genomic amplification of the MseI-1 sequence produced a single product of the expected size in both male and female samples (129 bp), whereas for MseI-2 at least five bands were obtained from male samples (188, 342, 500, 650, and 955 bp; Genbank acc. n. HR714301–HR714304) and no products in female samples. The five amplicons obtained from the male sample are imperfect tandem-repeats of the expected 188 bp MseI-2 amplicon (Fig. 2B). Southern blot analysis showed that this 188 bp portion of the MseI-2 sequence is a male-enriched sequence, as, although multiple bands ranging from 200 to 600 bp were visible in both male and female samples, their intensity was greater in the male samples (Fig. 2C). Moreover, FISH to mitotic chromosomes confirmed that the 188 bp sequence is located on the Y chromosome and on the pericentromeric region of autosome 2 (Fig. 2D).

**Figure 3:**
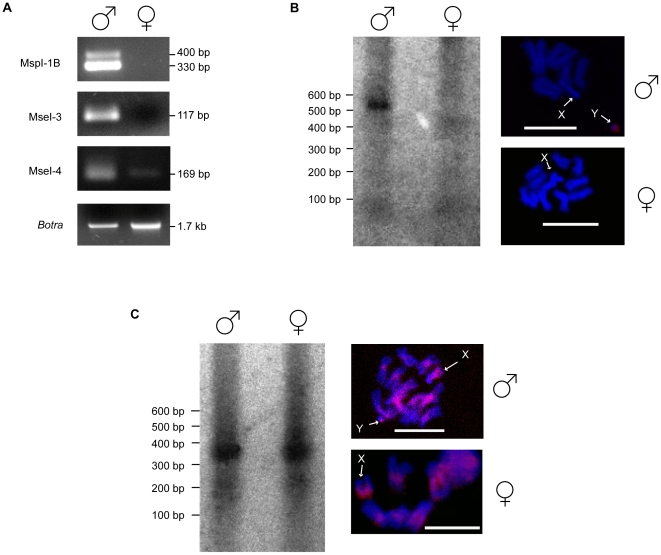
The *B. oleae* Y chromosome contains large interchromosomal duplications harbouring *importin-4* gene fragments. A) PCR amplifications on male and female genomic DNA from the Israel Hybrid strain using primers designed on the MspI-1B, MseI-3 and MseI-4 RDA clones. As a control, *Botransformer* (*Botra*) was amplified from the same samples. B–C) The Y-specificity of the MspI-1B and MseI-3 clones was verified using Southern blot analyses and fluorescence in situ hybridization to male and female *B. oleae* mitotic chromosomes preparations (scale bar 15 µm). For the Southern blot analyses male and female genomic DNA was digested with *MspI* or *MseI*, depending on the RDA clone used as probe.

Primers were also designed for the RDA sequences that are similar to insect importin-4 proteins, i.e. the MspI-1B, MseI-3 and MseI-4 clones (Table S2) and used to amplify male and female genomic DNA from the Israel Hybrid strain (Fig. 3A) in order to investigate the genomic distribution of these sequences. The MseI-4 primers produced the same product (169 bp) in both the male and female samples. However, despite using the same amount of genomic DNA template, the male *importin-4* amplification product was much more intense than that of the female. On the contrary, primers designed on MspI-1B clone amplified two bands only from male genomic DNA (330 bp and 400 bp). These two amplicons are closely related *importin-4*-like sequences sharing 72% identity. Similarly, primers designed on the MseI-3 RDA clone amplified a single *importin-4* band only in male individuals (117 bp).

The Y-specificity of the MspI-1B and the MseI-3 clones was tested using Southern blot analysis and in FISH. These analysis confirmed that the MspI-1B *importin-4* sequence is Y-specific, as it only hybridizes to a 550 bp fragment of male *Msp*I digested genomic DNA and only to the Y chromosome as shown in Fig. 3B. On the contrary, the MseI-3 *importin-4* clone hybridizes to a single band of about 300 bp in both male and female *Mse*I digested genomic DNA. In FISH analyses it hybridizes to the Y chromosome, to the highly heterochromatic long arm of the X chromosome and to the pericentromeric region of all the five autosomes (Fig. 3C).

### Isolation and characterization of a Y-specific *importin-4* gene fragment

**Figure 4:**
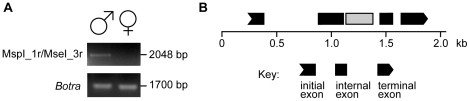
Presence of larger *importin-4* gene fragments on the *B. oleae* Y chromosome. **A**) Agarose gel electrophoresis of PCR products using the *Msp*I-1r and *Mse*I-3r primers sets on male and female genomic DNA from the Israel Hybrid strain. The *Botransformer* gene was amplified from the same samples as a positive control. **B**) Putative genomic organization of the Y specific *importin-4* gene fragments as predicted by GenScan analysis. The predicted exons are shown as black boxes. The BoY primer set was designed to amplify the exon region shown in grey.

To amplify a Y-specific *importin-4* gene fragment, longer than those present in the RDA clones, we used the primers designed to amplify the Y-specific *importin-4* MspI-1B and the MseI-3 sequences. Based on results of the BLASTX analyses of MspI-1B and MseI-3 RDA clones, we used the MspI-1Br and MseI-1r primer pair (Table S2). A single 2048 bp amplification product was obtained only from male-derived DNA (GenBank acc. n. HR714305) (Fig. 4A). BLASTN and BLASTX analyses of this 2048 bp fragment confirmed that it might be part of a gene similar to the *importin-4* genes of many insect species (Table 2). Using GenScan [26] it was also possible to predict a putative exon-intron structure for this gene fragment (Fig. 4B) and the protein encoded by the putative coding sequence is similar to Importin-4 proteins of different organisms, as suggested by the BLASTP analysis (Table 2).

**Table 2:** BLAST analyses of *importin-4* sequences identified in *B. oleae*.

Sequence	BLASTX^X^, BLASTN^N^ or BLASTP^P^ best hits	alignment length (bp)	e-value	maximum identity (%)
Y-specific sequence (2048 bp)	*Drosophila virilis* GJ12922^N^	196	4E-23	69
	*Aedes aegypti importin beta-4* (AAEL010698)^N^	217	2E-08	66
	*Culex quinquefasciatus importin-4* (CPIJ002334)^N^	225	7E-07	65
	*Drosophila wiilistoni* GK11274^X^	263	2E-60	47
	*Culex quinquefasciatus importin-4* (CPIJ002334)^X^	247	4E-55	44
	*Anopheles gambiae* AGAP004962^X^	262	3E-54	43
Y-specific *importin*-*4* (putative CDS)	*Drosophila wiilistoni* GK11274^P^	254	6E-47	42
	*Culex quinquefasciatus importin-4* (CPIJ002334)^P^	319	2E-44	35
	*Aedes aegypti importin beta-4* (AAEL010698)^P^	252	3E-44	40
autosomal *importin-4* gene (688 bp)	*Drosophila pseudoobscura* GA28523^N^	95	3E-12	78
	*Dropsophila melanogaster* CG32164^N^	64	3E-06	82
	*Apis melliphera* LOC412817^N^	43	4E-04	88
	*Culex quinquefasciatus importin-4* (CPIJ002334)^X^	37	1.0	75
	*Aedes aegypti importin beta-4* (AAEL010698)^X^	37	1.0	75
	*Drosophila virilis* GJ12922^X^	36	3.9	69

**Figure 5:**
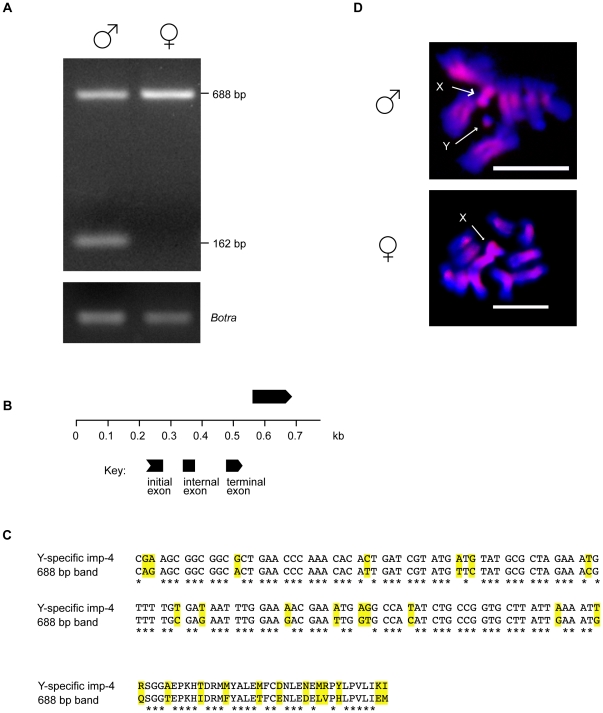
Presence of multiple copies of the *importin-4* gene fragment in the *B. oleae* genome. **A**) Agarose gel electrophoresis of PCR reactions using the BoY primer set on male and female genomic DNA from the Israel Hybrid strain. The *Botransformer* gene was amplified from the same samples as a positive control. B) Putative genomic organization of the 688 bp amplicon as predicted by GenScan analysis. The predicted exon of an *importin-4* gene is shown as a black box. **C**) Nucleotide and amino-acid alignment of putative coding portions of the Y-specific and non-Y specific *importin-4* gene fragments. Yellow shading represents areas of nucleotide and amino-acid changes. D) Fluorescence in situ hybridization on mitotic chromosomes spreads using the 688 bp band as probe (scale bar 15 µm). The upper panel shows male mitotic spreads, while the lower panel shows female mitotic spreads.

To test whether sequences closely related to the Y-specific *importin-4* gene fragments are also present on *B. oleae* autosomes or the X chromosome, primers were designed to amplify the putative third exon of the Y-specific *importin* gene fragment (light-grey in Fig. 4B). As expected, a 162 bp band was amplified only from male samples; however, an additional 688 bp band was amplified in both male and female samples (GenBank acc. n. HR714306) (Fig. 5A). BLASTN and BLASTX analyses of this 688 bp fragment showed that it is an additional *importin-4* gene fragment similar to the Y-specific one (Table 2). FISH using the 688 bp fragment as probe indicated that, in addition to the Y chromosome, similar sequences are located at the centromeric regions of all the autosomes and on the long arm of the X-chromosome (Fig. 5D).

The only predicted exon in the 688 bp sequence (Fig. 5B) codes for a putative 43 aa sequence that shares 62% identity with the putative Y-specific importin-4 protein (Fig. 5C). The Y-specific *importin-4* gene fragment and the one spread throughout the genome appear to be under neutral evolution (MEGA4 codon based Fisher's Exact test, P = 0.27) [28].

### Amplification of identified Y chromosome sequences is possible from different *B. oleae* samples, but not from other *Bactrocera* and *Ceratitis* species

To assess whether the Y sequences identified in this study are not restricted to the Israel Hybrid strain, the same set of primers (Table S2) designed on the MspI_1B, the MseI-2 and the MseI-3 RDA clones and on exon 3 of the Y-specific *importin-4* sequence were used to amplify male and female genomic DNA from individuals of different origins: i.e. from the Demokritos laboratory strain and from two wild populations, Jordan and Lebanon. As expected, amplicons were produced only from male-derived DNA for all the individuals analyzed (Fig. S2) and the amplification patterns were identical to those obtained in the Israel Hybrid strain.

To further investigate whether the *B. oleae* Y-specific or Y-enriched sequences are species-specific or genus-specific, the same set of primers listed above were used to amplify male and female genomic DNA from individuals from the most representative tephritid species. Four *Bactrocera* species (*B. dorsalis*, *B. invadens*, *B. correcta* and *B. cucurbitae*) and two *Ceratitis* species (*C. capitata* and *C. rosa*) were tested. Even using less stringent amplification conditions, it was not possible to recover any amplicon, neither in male nor female samples (Data not shown).

## Discussion

In the absence of any available sequence data, we used an RDA approach to investigate the Y chromosome of a tephritid fly, *Bactrocera oleae*, in order to derive information on its sequence content. The data provided insights on Y chromosome evolution in this Acalyptrate family, in which this chromosome plays an active role in the determination of male sex [8], [16].

The very small, highly heterochromatic Y chromosome of *B. oleae* is strewn with repetitive DNA sequences, the majority of which are also interdispersed in the pericentromeric regions of the autosomes. The Y chromosome appears to have accumulated small and large repetitive interchromosomal duplications. The large repetitive interchromosomal duplications harbour an *importin-4*-like gene fragment. Apart from these *importin-4*-like sequences, the other Y repetitive sequences are not shared with the X chromosome, suggesting the presence of molecular differentiation between these two chromosomes. Moreover, as the identified *B. oleae* Y specific or enriched regions were not detected on the Y-chromosomes of closely related tephritid species, we can infer a divergence in the repetitive nature of their sequence contents.

### The *B. oleae* Y chromosome harbours transposable elements, small duplications and longer gene fragments

Among the sequences isolated from the Y chromosome, two are transposable element fragments: an inactive *mariner* element and an *Osvaldo-like* retroelement. The enrichment of transposable elements on Y chromosome, particularly inactive *mariner* elements, is a feature already observed on the highly heterochromatic and largely degenerate Y chromosome of the related species, *C. capitata* [12]. Indeed, the accumulation of TEs in the Y heterochromatin, where recombination is strongly reduced, is an expected condition resulting from negative selection against the deleterious rearrangements caused by ectopic recombination between copies of the element in the euchromatin [34]. It has also been hypothesized that TEs have a major role in the degeneration of Y chromosomes by remodelling euchromatin into heterochromatic regions [35]. The presence of many transposable elements in the *B. oleae* Y chromosome is, therefore, a conserved feature, despite the diminutive size of this chromosome [6], [14].

Besides these expected repetitive sequences, the *B. oleae* Y chromosome appears to be also rich in sequences that are repeated on the Y chromosome itself and on the autosomes. The first sequence, which is similar to a portion of the *B. oleae Ovo* gene, is small and highly repeated in tandem arrays on the Y chromosome and on autosome 2. The second sequence is longer, harbours a fragment of an *importin-4*-like gene, and is also present on the pericentromeric regions of all the autosomes. The first type of duplication may be the result of an accumulation of smaller repetitions, while the second duplication may be the consequence of the spread of large blocks across the genome, particularly across highly heterochromatic regions, such as the pericentromeric regions. This pattern follows the proposed model of pericentromeric duplications first described in humans [36], that predicts an initial step of transposition seeding, caused by smaller duplications, followed by pericentromeric exchange of longer sequences [37].

The paralogous sequences of the larger regions, containing the *importin-4*-like gene fragment, present on the Y and on the other chromosomes, were shown to have evolved at neutral rates (codon based Fisher's Exact test). Applying the rate of 1% nucleotide divergence per Myr [38], [39] on both the Y-specific and autosomal *importin-4* sequences and on the *Ovo* related sequences, it was possible to calculate that these segmental duplications occurred respectively about 6 and 10 Mya, long after the split of Tephritidae and Drosophilidae, 80–100 Mya [40], [41], and, within the family Tephritidae, after the divergence of the *Bactrocera* and the *Ceratitis* genera 31.5 Mya [40]. Based on these data it appears that the enrichment of such sequences on the Y chromosome occurred relatively late during Acalyptrate evolution and that the presence of these sequences on the Y chromosome is probably limited to the *Bactrocera* genus, or perhaps to the species *B. oleae*. In agreement with this hypothesis, it was not possible to isolate *importin-4* related sequences from the Y chromosomes of four other *Bactrocera* species (*B. dorsalis*, *B. invadens*, *B. correcta* and *B. cucurbitae*) and of two *Ceratitis* species (*C. capitata* and *C. rosa*). However,we cannot exclude that this lack of amplification could be due to the sequence divergence of the primer regions, rather than a complete absence of such sequences in the genomes.

It has been observed that segmental duplications are usually involved in the multiplication of gene sequences in duplicated regions [36], [42], [43], [44]. This phenomenon was also observed in the *B. oleae* Y chromosome: the *importin-4* gene fragment was shown to be repeated on the Y chromosome, as well as being repeated also on the X chromosomes and the autosomes. In order to explain the persistent presence of such duplications on the Y chromosomes, it has been hypothesized that they are a reservoir for the maintenance of gene integrity. As the Y is a non-recombining chromosome, the mechanism proposed to maintain the identity of the repeat copies is the non-reciprocal transfer of sequences from one homologous locus to another. A similar mechanism could also be applied to the *B. oleae* Y chromosome, particularly to the *importin-4* gene fragments. This hypothesis, however, implies that the integrity of such sequences is necessary for the Y chromosome, or that they exert some structural function or some other molecular function. Genes of the *importin* family encode nuclear import receptors, which recognize the nuclear localization signal of protein cargo as α/β heterodimers [45]. In particular, *importin* β-like proteins, of which *importin-4* is a member, import SR proteins into the nucleus as they recognize the RS (arginine-serine rich) domain as a nuclear localization signal [46], [47], [48], [49], [50]. It is noteworthy that the *Bo-transformer* gene [16], the key component of the *B. oleae* sex-determination molecular cascade, is a member of the SR protein superfamily. It has also recently been demonstrated that an *importin-4* gene product is involved in the nuclear shuttling of the human male-determining factor protein, SRY [51]. In *B. oleae*, as in other Tephritidae species, the Y chromosome is known to be necessary to determine the male sex and a postulated Y-linked male-determining factor (M) directly or indirectly blocks the regulatory functions of the *Bo-transformer* gene [2], [8], [52]. However, even knowing the putative functions of importin-4 proteins, we cannot exclude that, once transposed on the *B. oleae* Y chromosome, the *importin-4* gene lost its original functions. Autosome-to-Y transposition is often followed by the duplication, exon pruning and degeneration of the transposed genes [53]. The degenerate genes and pseudogenes present in the repetitive regions of the human and *D. melanogaster* Y chromosomes are no longer considered simply ‘junk’ DNA, as they have assumed new functions such as stabilizing mRNA or controlling gene expression through the iRNA mechanism [54], [55]. Therefore, although the molecular functions of the general *importin* gene family are known, we cannot exclude that similar sequences on the *B. oleae* Y chromosome could have assumed novel functions during evolution. However, as the complete sequence of the *B. oleae* Y chromosome is not yet available, it is impossible to predict whether it contains putative complete functional *importin-4* genes or only pseudogenes that may have assumed new unpredictable functions.

### Conclusions and Outlook

Within the Tephritidae, the Y chromosomes share some conserved features: they are largely degenerate with much repetitive DNA [8], [11] and they are predicted to harbour an M factor that determines male development. Despite these apparently conserved features, the Y chromosomes display a high degree of variation in size and heterochromatin abundance, even among individuals of the same species [56]. The sequences here isolated from the *B. oleae* Y chromosome have no known related sequences on the Y chromosomes of other related genera, such as *C. capitata*: this may be indicative of a low conservation of sequence content. The high Y sequence-divergence within a taxonomic group belonging to the order Diptera was observed once the sequenced genomes of twelve different *Drosophila* species became available [57]. Among *Drosophila* species, the genetic content of the Y chromosome is fluid and Y-linked genes are poorly conserved; furthermore there are cases of wholesale replacement of the Y chromosome. It is noteworthy that divergence in repetitive sequences may cause reproductive isolation between nascent species [58]. Even if it is impossible to assess whether this is the case also for tephritid flies, as complete genome sequences are not yet available for any species, our data indicate that the Y chromosomes of tephritid species, or at least of the genera here considered, may have evolved independently. This suggestion has to be, however, proved and carefully tested.

The newly identified sequences of the *B. oleae* Y chromosome have no related sequences on the *Drosophila* sex chromosomes. This observation suggests that the tephritid sex chromosomes have a different evolutionary history with respect to the *Drosophila* X and Y. This suggestion is further supported by other experimental evidence: the *B. oleae* and, more generally, the tephritid Y chromosomes harbour the male determining factor, while that of *Drosophila* does not. By contrast, the *Drosophila* Y harbours many male-fertility genes, which are not located on the tephritid Y: this was first demonstrated in *C. capitata* [2], [8] and later in *B. oleae* [16]. The tephritid Y chromosome is extremely heteromorphic with respect to the X and displays a high degree of molecular divergence with it. The X-chromosomes of tephritid species are not homologous to those of *Drosophila*: they are completely heterochromatic and genes orthologous to the *Drosophila* X-linked genes are syntenic on different autosomes in different species [59], suggesting that the Tephritidae X chromosome is not syntenic to the *Drosophila* X chromosome. Therefore, even if the origin of the X chromosome in Diptera has been proposed to have occurred more than 260 Mya [57] as the *Drosophila* X chromosome is homologous to those of mosquito species of the genera *Anopheles* and *Aedes* [60], [61], the X chromosomes of Tephritids have evolved more recently and may have followed a distinct evolution.

Finally, as a practical application, we propose that the recent evolutionary history of tephritid sex chromosomes could facilitate the development of robust molecular diagnostic markers to discriminate species (i.e. BAR-coding) and sexes within a species [52], [62].

## Supporting Information

Figure S1 Schematic representations of regions that are similar between RDA clones and sequences found in Genbank using BLAST analyses. A) Schematic representation of nucleotide alignments between the *B. oleae Ovo* gene upstream region and three RDA clones (MspI-2B, MseI-1 and MseI-2). The MseI-1 and MseI-2 clones are partially similar to the same sequences in the *Ovo* promoter region. B) Schematic representation of amino-acid alignments between the *An. gambiae* AGAP006942 protein and three RDA clones (MspI-1B, MseI-3 and MseI-4).
(TIF)

Figure S2 Agarose gel electrophoresis of PCR reactions using the BoY primer set and primer sets designed to amplify the *Msp*I-1, *Mse*I-2 and *Mse*I-3 RDA clones. Male and female genomic DNA were used as template; *B. oleae* individuals of different origins were used (Israel Hybrid strain, Demokritos strain, wild individuals from Lebanon and Jordan). The *Botransformer* gene was amplified from the same samples as a positive control.
(TIF)

Table S1 Primers used for representational difference analysis (RDA).
(DOC)

Table S2 Primers used to amplify *Bactrocera oleae* male and female genomic DNA, and their respective annealing temperatures (Ta).
(DOC)

## References

[1] Sánchez L (2008). Sex-determining mechanisms in insects.. Int J Dev Biol.

[2] Saccone G, Pane A, Polito LC (2002). Sex determination in flies, fruitflies and butterflies.. Genetica.

[3] White I, Elson-Harris MM (1992). Fruit flies of economic significance : their identification and bionomics.

[4] Garcia-Martinez V, Hernandez-Ortiz E, Zepeta-Cisneros CS, Robinson AS, Zacharopoulou A (2009). Mitotic and polytene chromosome analysis in the Mexican fruit fly, *Anastrepha ludens* (Loew) (Diptera: Tephritidae).. Genome.

[5] Kounatidis I, Papadopoulos N, Bourtzis K, Mavragani-Tsipidou P (2008). Genetic and cytogenetic analysis of the fruit fly *Rhagoletis cerasi* (Diptera: Tephritidae).. Genome.

[6] Mavragani-Tsipidou P, Karamanlidou G, Zacharopoulou A, Koliais S, Kastritisis C (1992). Mitotic and polytene chromosome analysis in *Dacus oleae* (Diptera: Tephritidae).. Genome.

[7] Zhao JT, Frommer M, Sved JA, Zacharopoulou A (1998). Mitotic and polytene chromosome analyses in the Queensland fruit fly, *Bactrocera tryoni* (Diptera: Tephritidae).. Genome.

[8] Willhoeft U, Franz G (1996). Identification of the sex-determining region of the *Ceratitis capitata* Y chromosome by deletion mapping.. Genetics.

[9] Charlesworth B (2001). Genome analysis: More *Drosophila* Y chromosome genes.. Curr Biol.

[10] Anleitner JE, Haymer DS (1992). Y enriched and Y specific DNA sequences from the genome of the Mediterranean fruit fly, *Ceratitis capitata*.. Chromosoma.

[11] Zhou Q, Untalan PM, Haymer DS (2000). Repetitive A-T rich DNA sequences from the Y chromosome of the Mediterranean fruit fly, *Ceratitis capitata*.. Genome.

[12] Torti C, Gomulski LM, Moralli D, Raimondi E, Robertson HM (2000). Evolution of different subfamilies of mariner elements within the medfly genome inferred from abundance and chromosomal distribution.. Chromosoma.

[13] Montiel Bueno A, Jones O (2002). Alternative methods for controlling the olive fly, *Bactrocera oleae*, involving semiochemicals.. International Organization for Biological and Integrated Control of Noxious Animals and Plants West Palaearctic Regional Section (IOBC/WPRS) Bulletin.

[14] Mavragani-Tsipidou P (2002). Genetic and cytogenetic analysis of the olive fruit fly *Bactrocera oleae* (Diptera: Tephritidae).. Genetica.

[15] Lagos D, Ruiz MF, Sánchez L, Komitopoulou K (2005). Isolation and characterization of the *Bactrocera oleae* genes orthologous to the sex determining *Sex-lethal* and *doublesex* genes of *Drosophila melanogaster*.. Gene.

[16] Lagos D, Koukidou M, Savakis C, Komitopoulou K (2007). The *transformer* gene in *Bactrocera oleae*: the genetic switch that determines its sex fate.. Insect Mol Biol.

[17] Lisitsyn N, Wigler M (1993). Cloning the differences between two complex genomes.. Science.

[18] Drew AC, Brindley PJ (1995). Female-specific sequences isolated from *Schistosoma mansoni* by representational difference analysis.. Mol Biochem Parasitol.

[19] Donnison IS, Siroky J, Vyskot B, Saedler H, Grant SR (1996). Isolation of Y chromosome-specific sequences from *Silene latifolia* and mapping of male sex-determining genes using representational difference analysis.. Genetics.

[20] Fujisawa M, Hayashi K, Nishio T, Bando T, Okada S (2001). Isolation of X and Y chromosome-specific DNA markers from a liverwort, *Marchantia polymorpha*, by representational difference analysis.. Genetics.

[21] Tzanakakis ME, Economopoulos AP, Tsitsipis JA (1966). Improved artificial food media for larvae *Dacus oleae* (Gmelin) (Diptera:Tephritidae).. Z Angrew Entomol.

[22] Baruffi L, Damiani G, Guglielmino CR, Bandi C, Malacrida AR (1995). Polymorphism within and between populations of *Ceratitis capitata*: comparison between RAPD and multilocus enzyme electrophoresis data.. Heredity.

[23] Groot PC, van Oost BA (1998). Identification of fragments of human transcripts froma defined chromosomal region: representational difference analysis of somatic cell hybrids.. Nucleic Acids Res.

[24] Altschul SF, Gish W, Miller W, Myers EW, Lipman DJ (1990). Basic local alignment search tool.. J Mol Biol.

[25] Rozen S, Skaletsky H (2000). Primer3 on the WWW for general users and for biologist programmers.. Methods Mol Biol.

[26] Burge C, Karlin S (1997). Prediction of complete gene structures in human genomic DNA.. J Mol Biol.

[27] Poirot O, O'Toole E, Notredame C (2003). Tcoffee@igs: A web server for computing, evaluating and combining multiple sequence alignments.. Nucleic Acids Res.

[28] Tamura K, Dudley J, Nei M, Kumar S (2007). MEGA4: Molecular Evolutionary Genetics Analysis (MEGA) software version 4.0.. Mol Biol Evol.

[29] Southern EM (1975). Detection of specific sequences among DNA fragments separated by gel electrophoresis.. J Mol Biol.

[30] Robertson HM (1993). The *mariner* transposable element is widespread in insects.. Nature.

[31] Labrador M, Fontdevila A (1994). High transposition rates of *Osvaldo*, a new *Drosophila buzzatii* retrotransposon.. Mol Gen Genet.

[32] Pantazidis A, Labrador M, Fontdevila A (1999). The retrotransposon *Osvaldo* from *Drosophila buzzatii* displays all structural features of a functional retrovirus.. Mol Biol Evol.

[33] Khila A, El Haidani A, Vincent A, Payre F, Souda SI (2003). The dual function of *ovo*/*shavenbaby* in germline and epidermis differentiation is conserved between *Drosophila melanogaster* and the olive fruit fly *Bactrocera oleae*.. Insect Biochem Mol Biol.

[34] Charlesworth B, Langley CH, Sniegowski PD (1997). Transposable element distributions in *Drosophila*.. Genetics.

[35] Steinemann S, Steinemann M (2005). Y chromosomes: born to be destroyed.. BioEssays.

[36] Kirsch S, Weiss B, Miner T, Waterston R, Clark R (2005). Interchromosomal segmental duplications of the pericentromeric region on the human Y chromosome.. Genome Res.

[37] Eichler EE, Budarf ML, Rocchi M, Deaven LL, Doggett NA (1997). Interchromosomal duplications of the adrenoleukodystrophy locus: a phenomenon of pericentromeric plasticity.. Hum Mol Genet.

[38] Werman SD, Davidson EH, Britten RJ (1990). Rapid evolution in a fraction of the *Drosophila* nuclear genome.. J Mol Evol.

[39] Powell JR, Caccone A, Gleason JM, Nigro L (1993). Rates of DNA evolution in *Drosophila* depend on function and developmental stage of expression.. Genetics.

[40] Beverley SM, Wilson AC (1984). Molecular evolution in *Drosophila* and the higher Diptera II. A time scale for fly evolution.. J Mol Evol.

[41] Kwiatowski J, Skarecky D, Bailey K, Ayala FJ (1994). Phylogeny of *Drosophila* and related genera inferred from the nucleotide sequence of the Cu,Zn *Sod* gene.. J Mol Evol.

[42] Horvath JE, Schwartz S, Eichler EE (2000). The mosaic structure of human pericentromeric DNA: a strategy for characterizing complex regions of the human genome.. Genome Res.

[43] Luijten M, Wang Y, Smith BT, Westerveld A, Smink LJ (2000). Mechanism of spreading of the highly related neurofibromatosis type 1 (NF1) pseudogenes on chromosomes 2, 14 and 22.. Eur J Hum Genet.

[44] Samonte RV, Eichler EE (2002). Segmental duplications and the evolution of the primate genome.. Nat Rev Genet.

[45] Nakielny S, Dreyfuss G (1999). Transport of proteins and RNAs in and out of the nucleus.. Cell.

[46] Hedley ML, Amrein H, Maniatis T (1995). An amino acid sequence motif sufficient for subnuclear localization of an arginine/serine-rich splicing factor.. Proc Natl Acad Sci U S A.

[47] Cazalla D, Zhu J, Manche L, Huber E, Krainer AR (2002). Nuclear export and retention signals in the RS domain of SR proteins.. Mol Cell Biol.

[48] Kataoka N, Bachorik JL, Dreyfuss G (1999). Transportin-SR, a nuclear import receptor for SR proteins.. J Cell Biol.

[49] Lai MC, Lin RI, Tarn WY (2001). Transportin-SR2 mediates nuclear import of phosphorylated SR proteins.. Proc Natl Acad Sci U S A.

[50] Allemand E, Dokudovskaya S, Bordonné R, Tazi J (2002). A conserved *Drosophila* transportin-serine/arginine-rich (SR) protein permits nuclear import of *Drosophila* SR protein splicing factors and their antagonist repressor splicing factor 1.. Mol Biol Cell.

[51] Gontan C, Güttler T, Engelen E, Demmers J, Fornerod M (2009). *Exportin 4* mediates a novel nuclear import pathway for Sox family transcription factors.. J Cell Biol.

[52] Gabrieli P, Falaguerra A, Siciliano P, Gomulski LM, Scolari F (2010). Sex and the single embryo: early development in the Mediterranean fruit fly, *Ceratitis capitata*.. BMC Dev Biol.

[53] Gvozdev VA, Kogan GL, Usakin LA (2005). The Y chromosome as a target for acquired and amplified genetic material in evolution.. Bioessays.

[54] Premi S, Srivastava J, Epplen JT, Ali S (2010). AZFc region of the Y chromosome shows singular structural organization.. Chromosome Res.

[55] Kalmykova AI, Shevelyov YY, Dobritsa AA, Gvozdev VA (1997). Acquisition and amplification of a testis-expressed autosomal gene, SSL, by the *Drosophila* Y chromosome.. Proc Natl Acad Sci U S A.

[56] Willhoeft U, Franz G (1996). Comparison of the mitotic karyotypes of *Ceratitis capitata*, *Ceratitis rosa*, and *Trirhithrum coffeae* (Diptera: Tephritidae) by C-banding and FISH.. Genome.

[57] Bernardo Carvalho A, Koerich LB, Clark AG (2009). Origin and evolution of Y chromosomes: *Drosophila* tales.. Trends Genet.

[58] Ferree PM, Barbash DA (2009). Species-specific heterochromatin prevents mitotic chromosome segregation to cause hybrid lethality in *Drosophila*.. PLoS Biol.

[59] Stratikopoulos EE, Augustinos AA, Petalas YG, Vrahatis MN, Mintzas A (2008). An integrated genetic and cytogenetic map for the Mediterranean fruit fly, *Ceratitis capitata*, based on microsatellite and morphological markers.. Genetica.

[60] Nene V, Wortman JR, Lawson D, Haas B, Kodira C (2007). Genome sequence of *Aedes aegypti*, a major arbovirus vector.. Science.

[61] Zdobnov EM, von Mering C, Letunic I, Torrents D, Suyama M (2002). Comparative genome and proteome analysis of *Anopheles gambiae* and *Drosophila melanogaster*.. Science.

[62] Papathanos PA, Bossin HC, Benedict MQ, Catteruccia F, Malcolm CA (2009). Sex separation strategies: past experience and new approaches.. Malar J.

