# Supplementary figures and images for: Interchromosomal Duplications on the Bactrocera oleae Y Chromosome Imply a Distinct Evolutionary Origin of the Sex Chromosomes Compared to Drosophila
Source: PLoS One. 2011 Mar 7;6(3):e17747. doi: 10.1371/journal.pone.0017747 (PMC3049792; doi:10.1371/journal.pone.0017747)

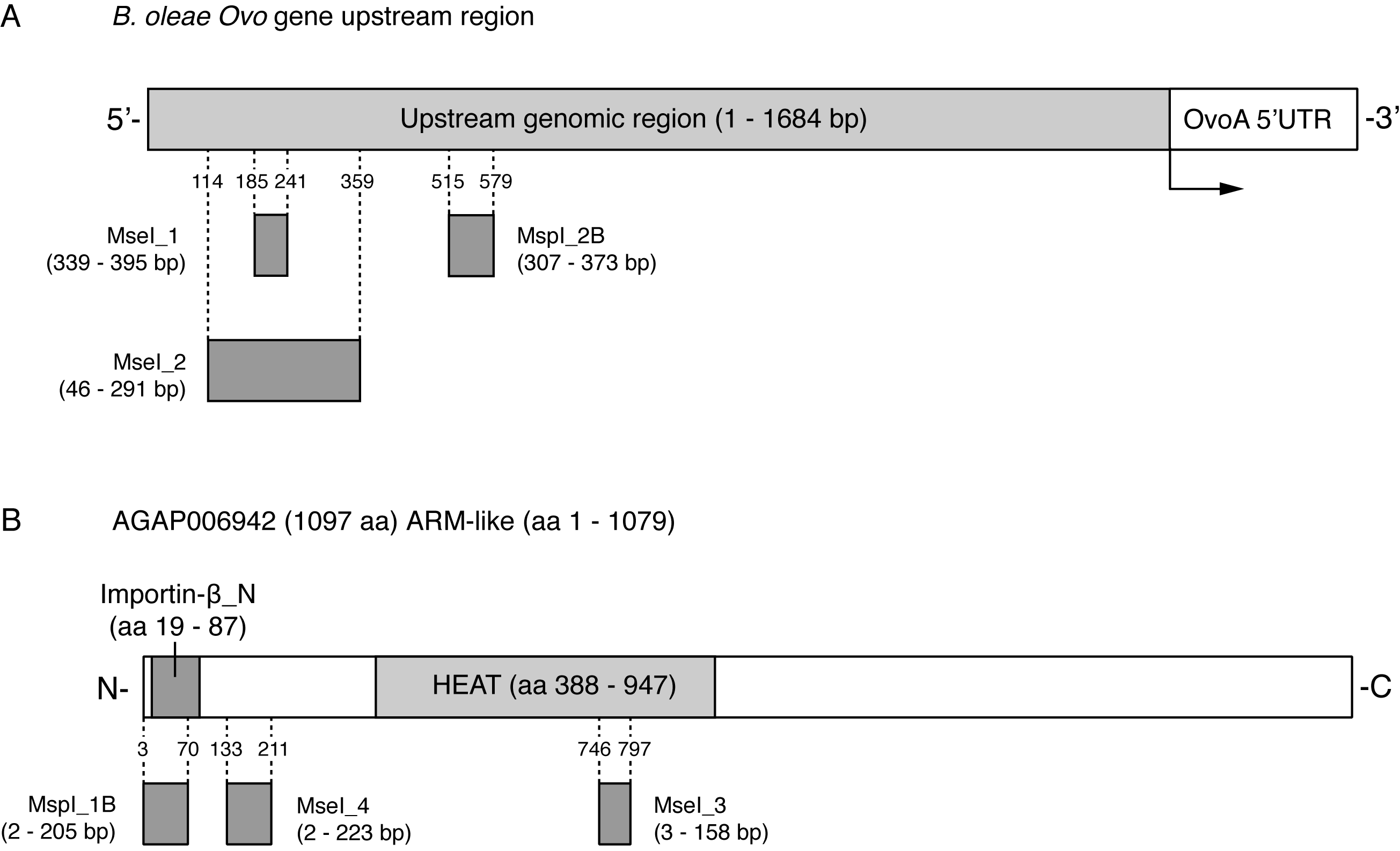

Supplement: Figure S1 — Schematic representations of regions that are similar between RDA clones and sequences found in Genbank using BLAST analyses. A) Schematic representation of nucleotide alignments between the B. oleae Ovo gene upstream region and three RDA clones (MspI-2B, MseI-1 and MseI-2). The MseI-1 and MseI-2 clones are partially similar to the same sequences in the Ovo promoter region. B) Schematic representation of amino-acid alignments between the An. gambiae AGAP006942 protein and three RDA clones (MspI-1B, MseI-3 and MseI-4). (TIF) [file pone.0017747.s001.tif]

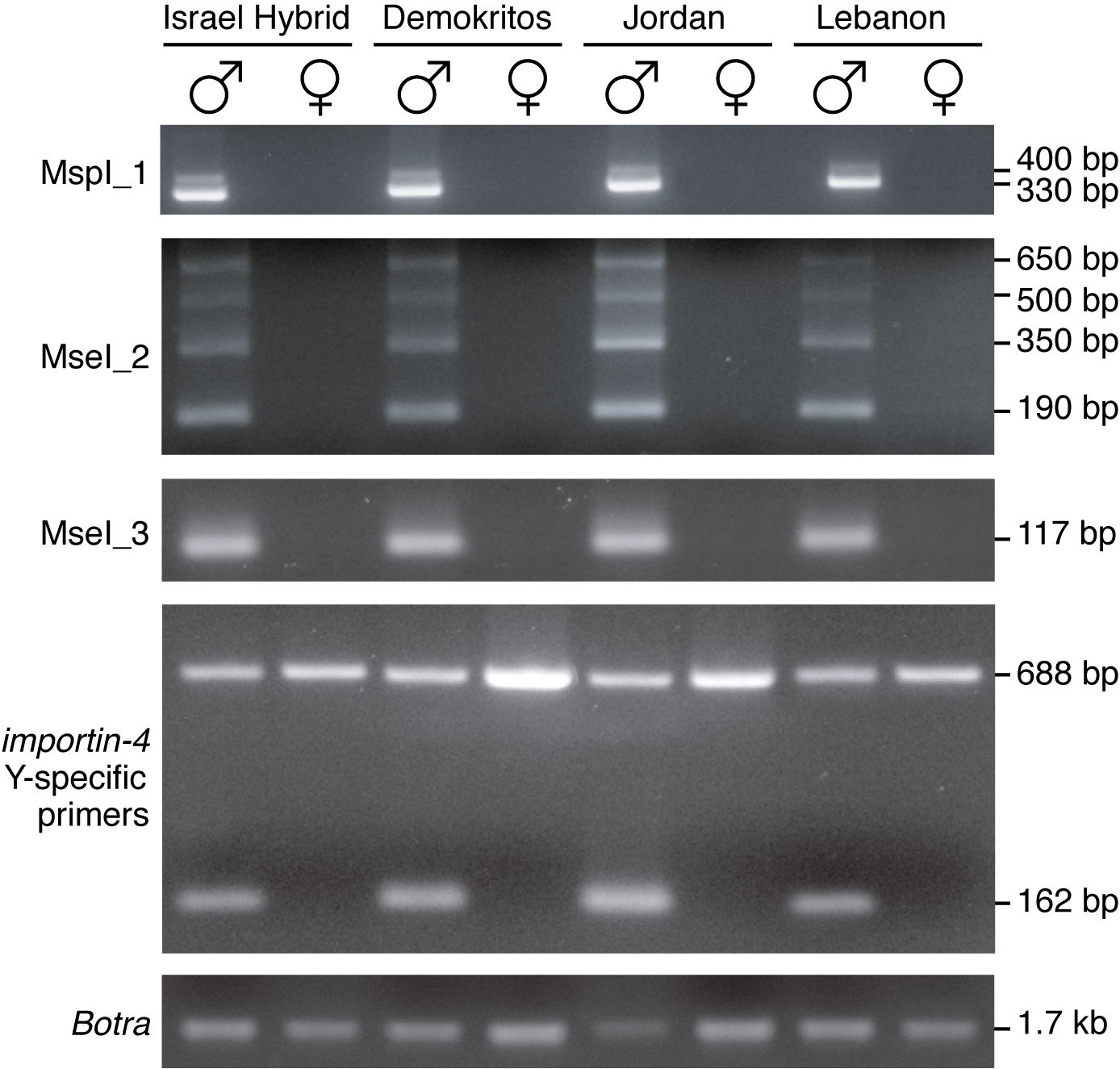

Supplement: Figure S2 — Agarose gel electrophoresis of PCR reactions using the BoY primer set and primer sets designed to amplify the MspI-1, MseI-2 and MseI-3 RDA clones. Male and female genomic DNA were used as template; B. oleae individuals of different origins were used (Israel Hybrid strain, Demokritos strain, wild individuals from Lebanon and Jordan). The Botransformer gene was amplified from the same samples as a positive control. (TIF) [file pone.0017747.s002.tif]
