# Supplementary material for: Interchromosomal Duplications on the Bactrocera oleae Y Chromosome Imply a Distinct Evolutionary Origin of the Sex Chromosomes Compared to Drosophila
Source: PLoS One. 2011 Mar 7;6(3):e17747. doi: 10.1371/journal.pone.0017747 (PMC3049792; doi:10.1371/journal.pone.0017747)
Supplement: Table S1 — Primers used for representational difference analysis (RDA). (DOC) [file pone.0017747.s003.doc]

**Table S1** Primers used for representational difference analysis (RDA). The R- series primers were used in the preparation of the amplicon representations, and the J- and N- series primers were used for odd and even hybridization-amplifications, respectively.

| Primer | Sequence (5' - 3') |
| --- | --- |
| R-Msp24 | AGCACTCTCCAGCCTCTCACCGCAC |
| R-Msp12 | CGGTGCGGTGAG |
| J-Msp24 | ACCGACGTCGACTATCCATGAACAC |
| J-Msp12 | CGGTGTTCATGG |
| N-Msp24 | AGGCAACTGTGCTACTCGAGGGAAC |
| N-Msp12 | CGGTTCCCTCGA |
| R-Mse24 | AGCACTCTCCAGCCTCTCACCGCAT |
| R-Mse12 | TAATGCGGTGAG |
| J-Mse24 | ACCGACGTCGACTATCCATGAACAT |
| J-Mse12 | TAATGTTCATGG |
| N-Mse24 | AGGCAACTGTGCTACTCGAGGGAAT |
| N-Mse12 | TAATTCCCTCGA |
