# Supplementary material for: Interchromosomal Duplications on the Bactrocera oleae Y Chromosome Imply a Distinct Evolutionary Origin of the Sex Chromosomes Compared to Drosophila
Source: PLoS One. 2011 Mar 7;6(3):e17747. doi: 10.1371/journal.pone.0017747 (PMC3049792; doi:10.1371/journal.pone.0017747)
Supplement: Table S2 — Primers used to amplify Bactrocera oleae male and female genomic DNA, and their respective annealing temperatures (Ta). (DOC) [file pone.0017747.s004.doc]

**Table S2** Primers used to amplify *Bactrocera oleae* male and female genomic DNA, and their respective annealing temperatures (Ta).

| Primer | Primer | Sequence | Ta (°C) |
| --- | --- | --- | --- |
| MspI-1B | Forward | 5’-ACGCTTGTAGGCCTCCTGTA | 58.0 |
|  | Reverse | 5’-CTGCCAGCTGAGGTAGTACG |  |
| MspI-2B | Forward | 5’-CACACACACACACACACACATC | 58.0 |
|  | Reverse | 5’-TTGTACTATATAGAGTCTGCGGCATT |  |
| MseI-1 | Forward | 5’-TCGCCATTATGTGGCATGTA | 58.0 |
|  | Reverse | 5’-CACACATGGAGTAGTAAGTCTACGG |  |
| MseI-2 | Forward | 5’-GTAGCTTCACGAAATGTTGCAT | 58.0 |
|  | Reverse | 5’-AACTGACAATTCGTGCCAAA |  |
| MseI-3 | Forward | 5’-ACGATCGGTTGTCGTAGAGG | 56.5 |
|  | Reverse | 5’-GCAGCGTTTATTCAGCTGTG |  |
| MseI-4 | Forward | 5’-GCGAGCTAGGTTCTACATTTGC | 58.0 |
|  | Reverse | 5’-GTGAACGGCACCAAAGAAGT |  |
| BoY | Forward | 5’-GCATCAGCTCTGCAATGAAT | 57.5 |
|  | Reverse | 5’-TTGTTGTGATTCAGAGCTTCG |  |
